# Evaluation of the Impact of a Tourniquet Training Program: A Cross-Sectional Study

**DOI:** 10.3390/ijerph20032742

**Published:** 2023-02-03

**Authors:** Valentín González-Alonso, María del Carmen Usero-Pérez, Raquel Seguido Chacón, Alicia Gómez de la Fuente, Jonathan Cortés-Martín, Raquel Rodríguez-Blanque, Juan Carlos Sánchez-García

**Affiliations:** 1 Departamento Simulación, Escuela Militar de Sanidad (EMISAN), Ministerio de Defensa, 28047 Madrid, Spain; 2 Departamento de Enfermería, Escuela Militar de Sanidad (EMISAN), 28047 Madrid, Spain; 3 Servicio de Sanidad de la Guardia Real, Ministerio de Defensa, 28048 Madrid, Spain; 4 Departamento de Enfermería, Facultad de Ciencias de la Salud, Universidad de Granada, 18071 Granada, Spain; 5 Hospital Clinico Universitario San Cecilio, 18016 Granada, Spain

**Keywords:** training, Stop the Bleed, tourniquet, bleeding control

## Abstract

Among the main preventable causes of death in the area of operations is external exsanguinating hemorrhage in the extremities, hence the importance of the tourniquet as a therapeutic tool in this type of injury and, therefore, of the training of personnel participating in international missions. The main objective of this study is to determine the impact of training in the application of this device. This is a quasi-experimental, prospective, cross-sectional study, carried out with 97 healthy volunteers, military personnel who perform their work in the Royal Guard barracks of El Pardo. The study was conducted between June 2019 and July 2021. The correct determination of the device placement site and the times of correct device placement were evaluated by determining whether there was blood flow using Doppler ultrasound measurements. Statistically significant results were obtained for application time (76.68 s to 58.06 s; *p* < 0.001), correct device placement (*p* < 0.001), and achievement of complete ischemia in the upper extremity (23.7% pretest vs. 24.7% post-test; *p* < 0.001). In the lower extremity, after training, longer application duration (43.33 s to 47.30 s) and lower ischemia achievement (59.8% pretest vs. 37.8% post-test) were obtained. Standardized and regulated training improves device application. More intensive training is necessary to obtain better results.

## 1. Introduction

Currently, it is estimated that 7 out of every 100 combat deaths could be prevented by the proper use of tourniquets [1]. The Committee of Tactical Combat Casualty Care (CoTCCC) [2] and the Prehospital Trauma Life Support (PHTLS) military edition [3] define that the main preventable causes of death in combat are exsanguinating hemorrhage, tension pneumothorax, and airway obstruction, in that order. 

More recently, Kelly et al. [4] published a retrospective analysis of data obtained from autopsies of combatants in the Iraq war and noted the following percentages of preventable battlefield deaths: external limb hemorrhage (33%) and airway obstruction (10–15%).

During the war conflicts in which the U.S. military has been involved, a considerable increase in survival among the wounded has been observed. During World War II, the percentage was 80%, in Vietnam it rose to 84%, and in the conflicts in Afghanistan and Iraq it was 90% [5].

Some of the factors associated with this increased probability of survival were physical readiness, proper marksmanship, tactics, and medical readiness [6]. 

Regarding medical preparedness, among the main preventable causes of death in the area of operations (AO) is external hemorrhage in extremities [4,5,7]. Some concepts of care of those wounded in combat have been designed that are collected in the doctrine Tactical Combat Casualty Care (TCCC or TC3) [6]. In this doctrine, some phases of combat casualty care are observed [8], such as Care Under Fire (CUF): the unit is under active hostile fire, and Tactical Field Care (TFC): the unit and casualty are still in a pre-hospital combat environment but not actively engaging the enemy. There is a safety period of about 15 min. Finally, Tactical Evacuation Care (TACEVAC/MEDEVAC): during this phase, the casualty is transported to a stable location for care.

Among the main preventable causes of death in combat is external exsanguinating hemorrhage. This is associated, in most cases, with severe vascular trauma caused by firearms or high explosives, also known as Improvised Explosive Devices (IEDs). These devices are detonated at a short distance from the combatant, causing severe vascular injuries with consequent blood loss, being responsible for a high percentage of combat casualties in modern war scenarios. The objective of every emergency service unit is to control blood loss early, to reduce the risk of death by exsanguination, to reduce the occurrence of consumption coagulopathy, acidosis, and hypothermia, to reduce fluid intake in resuscitation, and to prevent hypovolemic shock.

Current combat tactics developed in modern asymmetric warfare have increased the incidence of localized extremity trauma, which in combination with the knowledge that many of the deaths from combat injuries could have been avoided with the application of a tourniquet for effective hemorrhage control, provides a further reason for the use of tourniquets in current warfare [9]. Sebesta [10], who has detailed his experience as a surgeon in a military combat support hospital, notes that “tourniquets represent an essential treatment, based on the most recent experience in Iraq”.

This rationale is further reinforced by the circumstances influencing healthcare for the wounded on the battlefield. The real threat of the enemy, unfavorable environmental conditions, difficult terrain orography, longer evacuation times compared to civilian healthcare, austere logistics, and multiple wounded in a context of limited triage and treatment capabilities all support the timely use of tourniquets.

In CUF, it is essential that the injured person with bleeding in any of his limbs, or the closest companion if he is unable to do so, has sufficient knowledge and ability to apply the tourniquet to his limb in order to stop the bleeding [8].

Training has been a key element in increasing the survival of the wounded in the area of operations [11]. The training of all military personnel participating in international missions has significantly contributed to the decrease in mortality figures, especially in those techniques that address the main causes of preventable death in this environment (external bleeding, tension pneumothorax, and airway obstruction) [12,13,14].

During military operations, the first responder is considered the military personnel closest to the wounded, whose intervention will be essential to increase the chances of recovery of the wounded. Adequate training of this group will be a key factor in increasing the survival of the wounded in combat [15]. Training is essential for adequate medical care since insufficient training can lead to a high rate of errors in the treatment administered [16].

Military experience in the treatment of hemorrhage has been transferred to the civilian setting, where the necessary training is being provided, especially in intervention in mass casualty incidents [17,18,19], when civilian prehospital scenarios become austere or tactical, either due to a lack of material or excess casualties [20].

The American College of Surgeons (ACS) and the Department of Homeland Security (DHS) have encouraged and endorsed the use of tourniquets as part of the “Stop the Bleed” campaign. Both institutions have provided recommendations for training in tourniquet application by first responders to save the life of a victim suffering from external bleeding [21,22].

The Escuela Militar de Sanidad, a postgraduate training center of the Common Corps of the Ministry of Defense, has extensive experience in healthcare training in risk environments. In addition to its mission as a military teaching center, it has provided training in hemorrhage control in hostile environments to civilian institutions working in the field of emergencies (Municipal Emergency and Rescue Assistance Service—SAMUR, the Emergency Medical Service of Madrid—SUMMA, personnel of the Presidency of the Government, the Military School of Health, the Madrid Emergency Medical Service—SUMMA, personnel of the State Security Forces and Corps, personnel of the Presidency of the Government, Peace Observers, and war correspondents), with excellent results in the satisfaction surveys.

The Ministry of Defense, aware of the importance of standardizing the education and training of non-commissioned military personnel, has established guidelines for the instruction and training of non-commissioned personnel. One of the objectives is for military personnel to acquire competencies and capabilities that enable actions aimed at improving combat survivability [23].

Limb hemorrhage remains the leading cause of preventable death in the operating room [24]. Therefore, considering the importance of training in fundamental techniques such as tourniquet application, the aim of this study is to determine the impact of training in the application of this device.

## 2. Materials and Methods

### 2.1. Study Design

This is a quasi-experimental, prospective, cross-sectional study. The study was conducted in accordance with the Declaration of Helsinki for research involving human subjects and was approved by the Ethics Committee for drug research of the Hospital Central de la Defensa. This study has been approved by the Undersecretariat of Defense, which has declared it to be of high interest for the Armed Forces, in addition to having all the permits from the competent Military Authority to carry it out.

### 2.2. Setting

The study was carried out at the facilities of the Royal Guard. The Royal Guard is a military corps, belonging to the Spanish Armed Forces, dedicated to the protection of the King of Spain and the members of the Royal Family. Its installations are located in El Pardo (Madrid) and its personnel includes the three Armies and the Defense Corps.

The study was carried out from June 2019 to July 2021. The recruitment phase took place from June to November 2019 and the training phase was expected to start in January 2020 but due to the SARS-CoV-2 pandemic, this phase had to be postponed until January 2021. Data analysis was performed from March to June 2021. 

The device used for teaching and subsequent measurements of external bleeding control was the REF NEGRO model: COMMILN NOC 6515-33-2153184, developed by the company Manzanal Mamphel S.L., in its version 3.0.sal.

### 2.3. Participants

The eligibility criteria observed in the study were: military personnel carrying out their duties in the Royal Guard, aged between 18 and 50 years, regardless of their military category. All the volunteers had to have been declared fit in the annual examinations carried out by the health services of the Royal Guard. These examinations are carried out in accordance with Royal Decree 944/2001, of 3 August, which determines the psycho-physical fitness for service of Armed Forces personnel [25].

Once all those who met the inclusion criteria had been informed of the study, the military personnel who voluntarily wished to participate in the study were given the written documentation referring to the study and were summoned one week later. After this week of reflection and after asking the questions that they considered necessary, they signed the informed consent form.

In the informed consent and in the study documentation, it was emphasized that there would be no reward or sanction for personnel who participate or do not participate in the study.

Civilian personnel performing their duties in the Royal Guard, military personnel who were commissioned, or military personnel who, although declared fit for duty according to Royal Decree 944/2001, had some type of arteriovenous pathology, were excluded from the study. Also excluded were those participants who did not attend 100% of the training sessions and those who had not duly completed any of the documents required for the study.

### 2.4. Sociodemographic and Anthropometric Variables

The following parameters were measured as anthropometric data: Height and weight. Body weight (kg) was assessed with a calibrated scale and height (m) was measured using a calibrated metal rod. For the calculation of body mass index (BMI), the formula (BMI = weight in kg ÷ height m^2^) was used [26]. Participants were classified according to the WHO nutritional status [27]: underweight < 18.50 kg/m^2^, normal weight 18.50–24.99 kg/m^2^, overweight 25–29.99 kg/m^2^, and obesity ≥ 30.00 kg/m^2^ [26].

Sociodemographic data (age, gender, and military employment) were collected. As for the military employment variable, the military category held by the participant was collected, grouped into: soldier, corporal, corporal 1, corporal major, sergeant, and sergeant 1. Although there are other categories in the military scale, these are held by personnel above the age of the inclusion criterion. The military post was obtained from the personal file of each individual that was provided by the personnel unit of the Royal Guard.

### 2.5. Intervention-Related Variables

Intervention-related variables were collected, and knowledge was assessed using a questionnaire developed ad hoc by the investigators [28].

In addition, the tourniquet application time in the upper limb (UL) and in the lower limb (LL), both right and left, was recorded.

The correct application of the tourniquet was evaluated by two of the researchers, who measured the site of application of the tourniquet using a metal rod calibrated in cm, considering the correct application of the tourniquet between 4 and 6 cm above the site indicated as a wound.

The pain produced by the application of the tourniquet was assessed using the Visual Analog Scale (VAS) [29]. The VAS allows evaluating the pain described by the patient. It is graded from 0, no pain, to 10, severe pain.

After application of the tourniquet, a noninvasive Doppler ultrasound test was performed to estimate blood flow through the blood vessels, and the results were measured as “whether” or “not” blood flow remained through the blood vessels. Blood flow measurements were performed using the Hadeco Bidop ES-100 VII Doppler ultrasound (Hadeco, Inc., Chiyoda, Tokyo, Japan), by a vascular specialist physician, an expert in the use of Doppler ultrasound.

### 2.6. Bias

The investigators have not been able to determine whether there is a complacency bias in that the intervention was conducted by personnel in the senior military category or whether they could be considered vetted. Even so, the personnel who gathered the data concerning the variables were military personnel who do not work in the Royal Guard facilities. All participants were informed of the confidential nature of the data collected and that there was no sanction or reward based on the data recorded.

To avoid biases in data collection related to the variables to be considered, a rubric was used to evaluate both the location and the correct procedure for the application of the device.

### 2.7. Study Size

The sample size was not calculated, as the researchers sought the participation of all willing volunteer staff in order to obtain as much data as possible while contributing to their training.

### 2.8. Statistical Methods

The SPSS vs. 28.0 program for Windows (IBM Corporation, Armonk, NY, USA) was used for statistical analysis. The Student’s t test or the Mann–Whitney U test was used for the study of quantitative variables and the Chi-square test (or Fisher’s test if necessary) for the comparison of proportions. The results are expressed as mean, standard deviation, number, and/or percentage.

### 2.9. Intervention

The intervention consisted of an initial test of the use of the external bleeding control device, the tourniquet, in which each participant was asked to apply the tourniquet over clothing, in the proximal area of the upper extremity (UL) and lower extremity (LL). Subsequently, military personnel belonging to this unit were trained in the “Stop the Bleed^®^” course. The course was developed by the American College of Surgeons (ACS) and the ACS Committee on Trauma (ACS COT) and is certified free of charge. It lasted three hours, of which one hour was theoretical, in which the basics of external bleeding management were explained [30,31].

This was followed by the practical application of what was taught in the theoretical class, with a duration of two hours where the application of the tourniquet was performed.

After training and practice in the use of the tourniquet, a new practical test was performed in which each participant was again asked to apply the tourniquet over clothing, in the proximal UL and LL.

Before starting the procedure, a marker was used to mark the point where the pulse would be recorded with the Doppler ultrasound and the volunteer subject was instructed to indicate “ready” when the tourniquet application was finished, at which time the stopwatch was stopped. At this moment, the subject was shown a visual analog scale of pain and was asked to mark on it the pain he/she was experiencing at that moment. Once the application was finished, the absence of a pulse was confirmed by means of Doppler ultrasound at the previously marked point.

The evaluators were military medical personnel (physicians and nurses) with clinical experience in the teaching field studied, with more than 10 years of teaching experience, advanced training courses, and publications related to the practice evaluated, not belonging to the health services of the Royal Guard. Once the tourniquet was applied, they proceeded to collect the results using the ad hoc document created for this purpose. The variables of interest were the time taken to apply the tourniquet, the pain perceived during the procedure, and the confirmation of the presence or absence of a pulse after application of the tourniquet.

## 3. Results

Of the 120 students in the course, 97 finally participated in the study. The mean age of the participants was 30.76 years. Of the participants, 93.81% were male (*n* = 91), while 6.19% (*n* = 6) were female, and 87% were of normal weight and 13% were overweight. The physical condition of the sample showed that the overweight was due to excess muscle mass. Of the participants, 64.95% (63) were Private E2, 17.53% (17) were Corporals; 9.28% (9) were Sergeant; 1.03% (1) were Staff Sergeant; 4.12% (4) were Sergeants First Class 7.21% (7).

**Figure 1:**
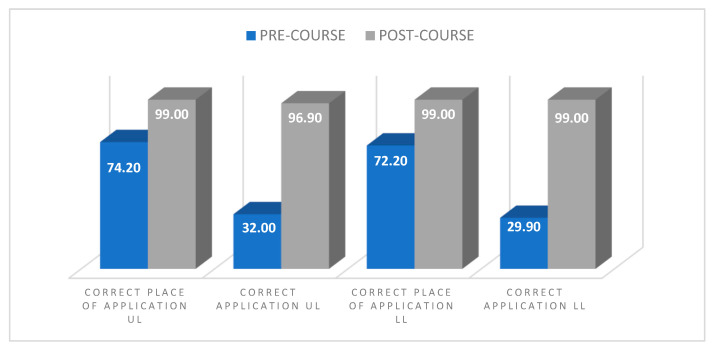
The percentage of results reported as “YES” is expressed.

Before and after training, the variables related to the correct application of the device and the correct application site on the upper and lower extremities were recorded, and the data are shown in Figure 1.

It was observed that a high proportion of the participants knew where to place the tourniquet (74.2% in UL, 72.2% in LL), however, they did not apply the tourniquet correctly.

Table 1 shows the pre-course and post-course results, depending on whether the device was applied to the upper or lower limb, for the variables time of application of the devices and the results of the VAS scale score, and the pre- and post-course questionnaire scores.

A statistically significant result was obtained in the analysis of correct tourniquet placement before and after training, in the UL and LL (*p* < 0.001). The same occurred in the study of learning the correct placement in the UL and LL (*p* < 0.001). Likewise, significant results were obtained for the time spent by the students in applying the device before and after training (UL *p* < 0.001; LL *p* = 0.021), as well as for the score obtained (*p* < 0.001). Finally, the analysis of pain manifested during the technique showed statistically significant results for both UL and LL (UL *p* < 0.001; LL *p* = 0.033) (Table 2).

The relationship of military employment category or sex of the participants with the results obtained was only found in the Doppler reading in LL (*p* = 0.025 as a function of sex and *p* = 0.007 as a function of military employment), with no significant results for the rest of the variables studied. 

## 4. Discussion

The training provided produced the expected results after completion of the training, which included tourniquet practice. Statistically significant results were observed in terms of tourniquet placement site and tourniquet placement time in both extremities, a fact that was also corroborated by the Doppler ultrasound. It should be noted that the results obtained in these studies corroborate the conclusions of Zwislewski et al. [32], who state that the personnel who perform the practical training are key to the acquisition of dexterity and skill in the use of the tourniquet and were in the same line as Ross et al. [18], who confirm the need for practical training for adequate hemorrhage control.

In this investigation, no relationship was observed between sociodemographic variables such as sex or employment of the participants and the results of training, except in the case of Doppler reading in LL after training. In the case of BMI, no relationship was observed with the training outcome.

There was a clear improvement in the results obtained in the time of application of the device in UL and in the points obtained in the evaluation of the practice after the training. Matthieu Boyé et al. [33] presented great results in the practice of tourniquet placement, although their study differs from ours in the duration of the training given, which was five months. However, ours was in the timeline of the study by Ross et al. [18], both of which were statistically significant with only 20 min of the theoretical class supported by simulations.

It is interesting to note that the analysis of the time used to apply the tourniquet shows that, after receiving training, the duration of application of this device in LL was longer (47.30 s) than before receiving training (43.33 s). This circumstance can be explained by the level of stress and nervousness of the participants during the pre- and post-test evaluations. Our data are better than those obtained by Schreckengaust, Littlejohn, and Zarow [34], the time for which using the SOFT-T tourniquet was 64 s and with the CAT, 57 s. However, the study by Heldenberg et al. [35] reflects a drastically shorter application time (CAT: 19 ± 7 s, SOFT-T: 24 ± 7 s). This difference may be because although both works used military personnel in training as a sample, the study with which we compared our results obtained its data from volunteers belonging to the Israeli Naval Special Warfare Unit (INSWU). The soldiers belonging to this Unit had extensive combat experience and advanced knowledge of tactical first aid.

Similarly, it was striking to observe how pain, both in the UL (pretest VAS = 4.48, post-test VAS = 5.31) and the LL (pretest VAS = 5.68, post-test VAS = 6.24), increased after training, a result that can be explained by the improvement in the performance of the practice by exerting greater pressure on the limbs after training.

Regarding placement in the correct location, the data provided in this study are similar to those obtained by Schreckengaust, Littlejohn, and Zarow [34]. In our study, a 99% hit rate was obtained, very similar to those obtained for CAT by Schreckengaust, Littlejohn, and Zarow [34], with 94%, however, in the SOFT-T application, it was slightly lower, with 87%.

Scott et al. [36] conducted their study in other military settings, such as pilot or medical personnel training, in which they found a significant improvement in outcomes after training with the Tactical Combat Casualty Care (TCCC) course, which includes training in tourniquet application. Their research found a significant improvement in theoretical knowledge as well as in good practices after training by 100% of the participants, data that agree with those obtained in our study.

This study was conducted during training in a single military unit (Royal Guard), so it would be interesting to compare the results with other units in which healthcare training in a combat environment is mandatory due to the activity performed, such as special operations units. It has been observed that personnel with this type of training have greater confidence in the possible care of those wounded in combat [37].

As a complement to this work, it would be interesting to obtain results of the satisfaction perceived by the students after the course by means of a survey. Other similar studies have obtained very positive results in the assessment made by the students [19].

Further research is needed in stress environments similar to those of combat, which could modify the results of our study [13].

## 5. Conclusions

As previously stated, hemorrhage is the main cause of preventable death in combat, and the training of personnel who can participate in combat and become first responders to a casualty is essential to reduce the number of casualties. In this work, it was shown that theoretical and practical training in bleeding control and tourniquet use is statistically significant and necessary.

The completion of a course such as “Stop the Bleed” by personnel who may participate in military conflicts improves the acquisition of knowledge and the execution of techniques such as the application of tourniquets.

Training programs such as the “TCCC” course or the “Stop the Bleed” course contribute to improving training in the correct treatment of external bleeding, helping to improve survival rates both in the combat zone and during assistance in multiple casualty incidents.

Considering the large number of military personnel who perform their duties in a war environment, we consider this training necessary to train first responders in the management of external bleeding.

During the study, none of the tourniquets suffered apparent deformation or damage to any of their components during application.

## Figures and Tables

**Table 1:** Results of time, VAS, and test score variables.

Variable	Mean	DT	Max	Min
Time of application UL pre-course (seconds)	76.68	31.25	196.00	26.97
Time of application UL post-course (seconds)	58.06	18.46	138.00	26.99
VAS UL pre-course (score)	4.48	2.15	9	0
VAS UL post-course (score)	5.31	2.13	9	0
Time of application LL pre-course (seconds)	43.33	13.12	84.18	15.91
Time of application LL post-course (seconds)	47.30	12.86	98.00	22.00
VAS LL pre-course (score)	5.68	2.45	9	0
VAS LL post-course (score)	6.24	2.01	9	0
Puntuation pre-course (score)	6.21	1.92	9.50	1
Puntuation post-course (score)	8.95	1.43	10	2

SD: standard deviation; LL: lower limb; VAS: Virtual Analog Scale; UL: upper limb.

**Table 2:** Results by sex, military category (employment), and BMI. Values in bold denote statistical significance (*p* < 0.05), Upper limb (UL), lower limb (LL).

Variable (Chi-Square)	Sex (*p*-Value)	Employment (*p*-Value)	BMI (*p*-Value)
Correct place of application UL pretest	0.159	0.189	0.215
Correct application of UL pretest	0.606	0.260	0.563
Pretest UL Doppler reading	0.128	0.356	0.686
Correct application UL pretest	0.417	0.656	0.605
Pretest UL Doppler reading	0.460	0.330	0.374
Doppler reading UL post-test	0.159	0.226	0.416
Correct place LL post-test	0.938	0.990	0.669
Correct application LL post-test	0.938	0.079	0.516
Doppler reading LL post-test	0.025	0.007	0.705
Correct place LL pretest	0.463	0.596	0.497
Correct place UL post-test	0.938	0.990	0.669
Correct application UL post-test	0.824	0.742	0.705

## Data Availability

Data are available on request from the corresponding author.
